# Towards the Description of the Genome Catalogue of *Pseudomonas* sp. Strain M1

**DOI:** 10.1128/genomeA.00146-12

**Published:** 2013-02-07

**Authors:** Pedro Soares-Castro, Pedro M. Santos

**Affiliations:** CBMA, Center of Molecular and Environmental Biology, Department of Biology, University of Minho, Campus de Gualtar, Braga, Portugal

## Abstract

*Pseudomonas* sp. strain M1 is a soil isolate with remarkable biotechnological potential. The genome of *Pseudomonas* sp. M1 was sequenced using both 454 and Illumina technologies. A customized genome assembly pipeline was used to reconstruct its genome sequence to a single scaffold.

## GENOME ANNOUNCEMENT

*Pseudomonas* sp. strain M1, isolated from the Rhine River (1), is able to utilize several toxic and/or recalcitrant compounds, such as myrcene (1, 2), citral, citronellol, phenol (3–5), chlorophenols, and benzene (5), as its sole carbon and energy sources. However, the molecular mechanisms of M1 strain that are associated with the utilization of those (and other) less common carbon sources are still poorly known. To set the proper background for exploring the biotransformation potential of *Pseudomonas* sp. M1, its genome was sequenced using both 454 FLX and Illumina Genome Analyzer IIx next-generation sequencing technologies. The 454 FLX sequencing technology yielded a 264,177 single-read data set with an average read length of 523 bp, whereas the Illumina technology was used to produce two 50-bp read-length data sets: (i) 5,303,579 pair-end reads with an estimated insert size of about 320 bp; and (ii) 5,478,608 mate-paired reads with an estimated insert size of about 5,200 bp. The removal of adapter sequences and quality trimming were performed in all data sets prior to *de novo* assembly. To reconstruct the genome of *Pseudomonas* sp. M1, a customized pipeline was set, based on preliminary comparative trials using different genome assemblers. First, 454 FLX single reads were assembled with Newbler v2.6 (6), generating 379 contigs (minimum contig size of 1,000 bp) with a total size of 6,860,386 bp. Second, the 454 FLX-generated contigs were used as a genome backbone to produce eight scaffolds using both Illumina libraries in SSPACE v2.0 (7). The scaffolds we obtained included over 200 gaps, which were significantly filled by combining local alignment with GapFiller (8) and GapCloser (9). To obtain a more contiguous and accurate genome sequence, a further sequential run of SSPACE (7), GapFiller (8) and GapCloser (9), and Anchor v0.3.1 (http://www.bcgsc.ca/platform/bioinfo/software/anchor) was done, resulting in a single scaffold representing the genome of *Pseudomonas* sp. M1. Further genome sequence accuracy was improved by manual curation (based on read alignment analysis) and Sanger sequencing to confirm sequence accuracy and to close different sequence gaps. Nonetheless, nine repeat-rich regions were not fully resolved. As a whole, the current draft of the *Pseudomonas* sp. M1 genome is composed of nine contigs organized in a single scaffold, with a total size of 6,958,606 bp (including 1,753 N's), with an estimated G+C content of 67.3%. This genome sequence was annotated using Prokka v1.5.2 (http://www.vicbioinformatics.com/software.prokka.shtml) and deposited at DDBJ/EMBL/GenBank. The annotated genome includes 6,053 coding sequences (CDSs), 12 rRNAs (four copies each of 5S, 16S, and 23S rRNA), 66 tRNAs, and 804 hypothetical proteins.

Further inspection of the genome of *Pseudomonas* sp. M1 revealed the presence of a significant number of biotechnologically interesting enzymes (e.g., 74 oxygenases/hydroxylases) whose functionality may be fine-tuned using systems and/or synthetic biology approaches.

### Nucleotide sequence accession numbers.

This Whole Genome Shotgun project (Bioproject: PRJNA62721) has been deposited at DDBJ/EMBL/GenBank under the accession number ANIR00000000. The version described in this article is the first version, ANIR01000000.

## References

[1] Iurescia S Marconi AM Tofani D Gambacorta A Paternò A Devirgiliis C Van Der Werf MJ Zennaro E 1999 Identification and sequencing of beta-myrcene catabolism genes from *Pseudomonas* sp. strain M1. Appl. Environ. Microbiol. 65:2871–2876 10388678 10.1128/aem.65.7.2871-2876.1999 PMC91431

[2] Santos PM Sá-Correia I 2009 Adaptation to beta-myrcene catabolism in *Pseudomonas* sp. M1: an expression proteomics analysis. Proteomics 9:5101–5111 19798672 10.1002/pmic.200900325

[3] Santos PM Mignogna G Heipieper HJ Zennaro E 2002 Occurrence and properties of glutathione S-transferases in phenol-degrading *Pseudomonas* strains. Res. Microbiol. 153:89–98 11900268 10.1016/s0923-2508(01)01293-1

[4] Santos PM Roma V Benndorf D Von Bergen M Harms H Sá-Correia I 2007 Mechanistic insights into the global response to phenol in the phenol-biodegrading strain *Pseudomonas* sp. M1 revealed by quantitative proteomics. Omics J. Integr. Biol. 11:233–251 10.1089/omi.2007.0009 17883337

[5] Santos PM Sá-Correia I 2007 Characterization of the unique organization and co-regulation of a gene cluster required for phenol and benzene catabolism in *Pseudomonas* sp. M1. J. Biotechnol. 131:371–378 17826858 10.1016/j.jbiotec.2007.07.941

[6] Margulies M Egholm M Altman WE Attiya S Bader JS Bemben LA Berka J Braverman MS Chen YJ Chen Z Dewell SB Du L Fierro JM Gomes XV Godwin BC He W Helgesen S Ho CH Ho CH Irzyk GP Jando SC Alenquer ML Jarvie TP Jirage KB Kim JB Knight JR Lanza JR Leamon JH Lefkowitz SM Lei M Li J Lohman KL Lu H Makhijani VB McDade KE McKenna MP Myers EW Nickerson E Nobile JR Plant R Puc BP Ronan MT Roth GT Sarkis GJ Simons JF Simpson JW Srinivasan M Tartaro KR Tomasz A Vogt KA Volkmer GA Wang SH Wang Y Weiner MP Yu P Begley RF Rothberg JM 2005 Genome sequencing in microfabricated high-density picolitre reactors. Nature 437:376–380 16056220 10.1038/nature03959 PMC1464427

[7] Boetzer M Henkel CV Jansen HJ Butler D Pirovano W 2011 Scaffolding pre-assembled contigs using SSPACE. Bioinformatics 27:578–579 21149342 10.1093/bioinformatics/btq683

[8] Boetzer M Pirovano W 2012 Toward almost closed genomes with GapFiller. Genome Biol. 13:R56 22731987 10.1186/gb-2012-13-6-r56 PMC3446322

[9] Li R Fan W Tian G Zhu H He L Cai J Huang Q Cai Q Li B Bai Y Zhang Z Zhang Y Wang W Li J Wei F Li H Jian M Li J Zhang Z Nielsen R Li D Gu W Yang Z Xuan Z Ryder OA Leung FC Zhou Y Cao J Sun X Fu Y Fang X Guo X Wang B Hou R Shen F Mu B Ni P Lin R Qian W Wang G Yu C Nie W Wang J Wu Z Liang H Min J Wu Q Cheng S Ruan J Wang M Shi Z Wen M Liu B Ren X Zheng H Dong D Cook K Shan G Zhang H Kosiol C Xie X Lu Z Zheng H Li Y Steiner CC Lam TT-Y Lin S Zhang Q Li G Tian J Gong T Liu H Zhang D Fang L Ye C Zhang J Hu W Xu A Ren Y Zhang G Bruford MW Li Q Ma L Guo Y An N Hu Y Zheng Y Shi Y Li Z Liu Q Chen Y Zhao J Qu N Zhao S Tian F Wang X Wang H Xu L Liu X Vinar T Wang Y Lam T-W Yiu S-M Liu S Zhang H Li D Huang Y Wang X Yang G Jiang Z Wang J Qin N Li L Li J Bolund L Kristiansen K Wong GK-S Olson M Zhang X Li S Yang H Wang J Wang J 2010 The sequence and de novo assembly of the giant panda genome. Nature 463:311–317 20010809 10.1038/nature08696 PMC3951497

